# COG5-congenital disorder of glycosylation diagnosed by whole genome sequencing in siblings with unexplained optic atrophy, macular atrophy, and developmental delay: case report

**DOI:** 10.3389/fneur.2026.1840802

**Published:** 2026-06-01

**Authors:** Katherine Granger, Catherine Do, Catherine Quindipan, Ryan Schmidt, Mark S. Borchert, Aaron Nagiel, Melinda Y. Chang

**Affiliations:** 1 Department of Surgery, Vision Center, Children’s Hospital Los Angeles, Los Angeles, CA, United States; 2 Department of Ophthalmology, Roski Eye Institute, Keck School of Medicine, University of Southern California, Los Angeles, CA, United States; 3 Department of Pathology and Laboratory Medicine, Children’s Hospital Los Angeles, Los Angeles, CA, United States; 4 Department of Pathology, Keck School of Medicine, University of Southern California, Los Angeles, CA, United States

**Keywords:** case report, congenital disorders of glycosylation, developmental delay, optic atrophy, retinal dystrophy

## Abstract

**Introduction:**

*COG5*-related congenital disorder of glycosylation (COG5-CDG) is a rare autosomal recessive metabolic disorder with variable neurologic and ophthalmologic involvement. We report two siblings presenting with optic atrophy, macular atrophy, and neurodevelopmental delay; these three phenotypic manifestations have not, to our knowledge, been combined together in previously described individuals with COG5-CDG. The genetic disorder was only diagnosed through whole genome sequencing (WGS) after panel-based exome testing was unrevealing.

**Clinical findings:**

Both siblings presented with early-onset severe visual impairment, bilateral optic atrophy, central macular atrophy, sensory nystagmus, and strabismus in the setting of global developmental delay. Additional features included polymicrogyria and hypotonia (Case 1) and microcephaly and autism spectrum disorder (Case 2).

**Diagnoses and outcomes:**

After repeated non-diagnostic inherited retinal gene panels, clinical quad WGS identified compound heterozygous variants in *COG5* in both siblings: a paternally inherited pathogenic frameshift variant (c.1415dup) and a maternally inherited deep intronic variant (c.417 + 4779A>G) with high SpliceAI-predicted splicing impact. Visual function has remained relatively stable over 5 years of follow-up.

**Conclusion:**

These cases expand the phenotypic spectrum of COG5-CDG to include concurrent optic nerve and macular involvement with neurodevelopmental impairment and highlight the essential role of WGS in diagnosing complex neuro-ophthalmologic presentations when targeted genetic testing is nondiagnostic.

## Introduction

Congenital disorders of glycosylation (CDG) are a group of inherited metabolic disorders caused by impaired glycosylation of proteins and lipids. These conditions commonly affect the central nervous system and are associated with developmental delay, hypotonia, seizures, and growth abnormalities, often in the setting of broader multisystem involvement (1–3). Neurologic features frequently dominate the clinical presentation and may evolve over time, contributing to delayed recognition of an underlying etiology (1–3).

Several CDG subtypes result from defects in the conserved oligomeric Golgi (COG) complex, which is required for normal Golgi trafficking and proper localization of glycosylation enzymes (4–6). One such subtype, CDG type IIi (COG5-CDG) leads to a rare autosomal recessive condition characterized by global developmental delay, hypotonia, variable structural brain abnormalities, autistic behaviors, and liver disease including cirrhosis (7–9). Ophthalmologic findings have been reported inconsistently. Strabismus has been reported most frequently, and two cases of cortical blindness were described in children with severe neurologic abnormalities including diffuse brain atrophy on magnetic resonance imaging (MRI) and progressive microcephaly (7–9). More recently, early-onset retinal degeneration, foveal hypoplasia, and optic nerve atrophy have been reported in association with pathogenic *COG5* variants, suggesting that visual system involvement may be more extensive than previously recognized (9–11). The three related individuals with early-onset severe retinal degeneration did not have neurocognitive impairment (10).

Children with ophthalmologic and neurodevelopmental features frequently undergo prolonged diagnostic evaluations (12, 13). Targeted genetic testing is often pursued early in evaluation, yet nondiagnostic results are common. Genome-wide sequencing approaches can be particularly informative in this setting, especially when disease-causing variants lie outside regions typically assessed by panel-based testing (14–16).

Herein we report two siblings with developmental delay and severe visual impairment characterized by bilateral optic atrophy, macular involvement, and nystagmus, in whom genetic testing was initially unrevealing. They were ultimately diagnosed with COG5-related congenital disorder of glycosylation with compound heterozygous variants, one intronic and one exonic, through whole genome sequencing (WGS). These cases expand the phenotype of COG5-CDG and illustrate the value of genome-wide testing in children with unexplained neuro-ophthalmologic disease.

## Case description

Two siblings, a 17-year-old female (Case 1) and her 7-year-old brother (Case 2), were referred for evaluation of longstanding developmental delay and severe visual impairment. They were born to non-consanguineous parents of African American, Native American, and mixed European ancestry. Family history was notable for a father with sickle cell trait and myopia, a paternal half-sister with attention-deficit hyperactivity disorder, and extended maternal and paternal relatives with intellectual disability. An older brother was developmentally typical and had normal visual function.

### Clinical case descriptions

#### Case 1

Case 1 is a 17-year-old female with global developmental delay, nystagmus, optic atrophy, polymicrogyria, scoliosis, and bilateral macular atrophy. She was born at 39 weeks’ gestation via repeat cesarean delivery with a birth weight of 5 lb. 11 oz. Prenatal history was notable for hyperemesis gravidarum. Prenatal screening and fetal ultrasounds were reported as normal, and there were no known teratogenic exposures.

Developmental delay was identified by the family’s pediatrician in infancy. She sat independently at 9 months, spoke her first word at approximately 18 months, and walked at 23 months. She was enrolled in special education and received physical therapy, occupational therapy, adaptive physical education, and orientation and mobility training, as well as orthopedic surgery for scoliosis.

At 9 years of age, brain MRI with and without contrast performed at an outside institution demonstrated right frontal polymicrogyria. Repeat MRI of the orbits, face, and neck performed at 10 years old demonstrated bilateral optic nerve and optic chiasm atrophy. At approximately 11 years of age, she was evaluated by an outside ophthalmologist for suspected visual impairment. Bilateral optic atrophy and macular scarring were documented at that time. Because of difficulty with compliance during the examination, a reliable estimate of visual acuity could not be obtained. Glasses had been prescribed previously but were not tolerated.

After her ophthalmologic assessment, she was evaluated by metabolic genetics for global developmental delay, bilateral optic atrophy with macular scarring, nystagmus, scoliosis, congenital hypotonia, and social and communication deficits. Due to the findings of developmental delay and hypotonia, an inherited retinal disease focused exome panel, including analysis of *ACO2*, was performed but returned negative results.

At 12 years of age, she was first evaluated by neuro-ophthalmology at Children’s Hospital Los Angeles (CHLA). Her visual acuity was 1/60 in each eye. Pupil reactivity was weak and there was no relative afferent pupillary defect (RAPD). Stereopsis was nil, and ductions were full. She had a V-pattern exotropia measuring 30 PD at near in primary gaze. Conjugate, pendular, horizontal nystagmus was present. Fundus examination demonstrated severe bilateral optic atrophy. There was no comment on macular pathology. Mild anisometropia with hypermetropic astigmatism was noted, and refractive correction was not expected to improve visual function. She was unable to cooperate with ophthalmic imaging at this time. She underwent ocular focused exome sequencing at CHLA which was unrevealing, as no clinically significant variants or variants of uncertain significance were identified (Supplementary material). She was then referred to genetic counseling for consideration of additional genetic testing.

**Figure 1:**
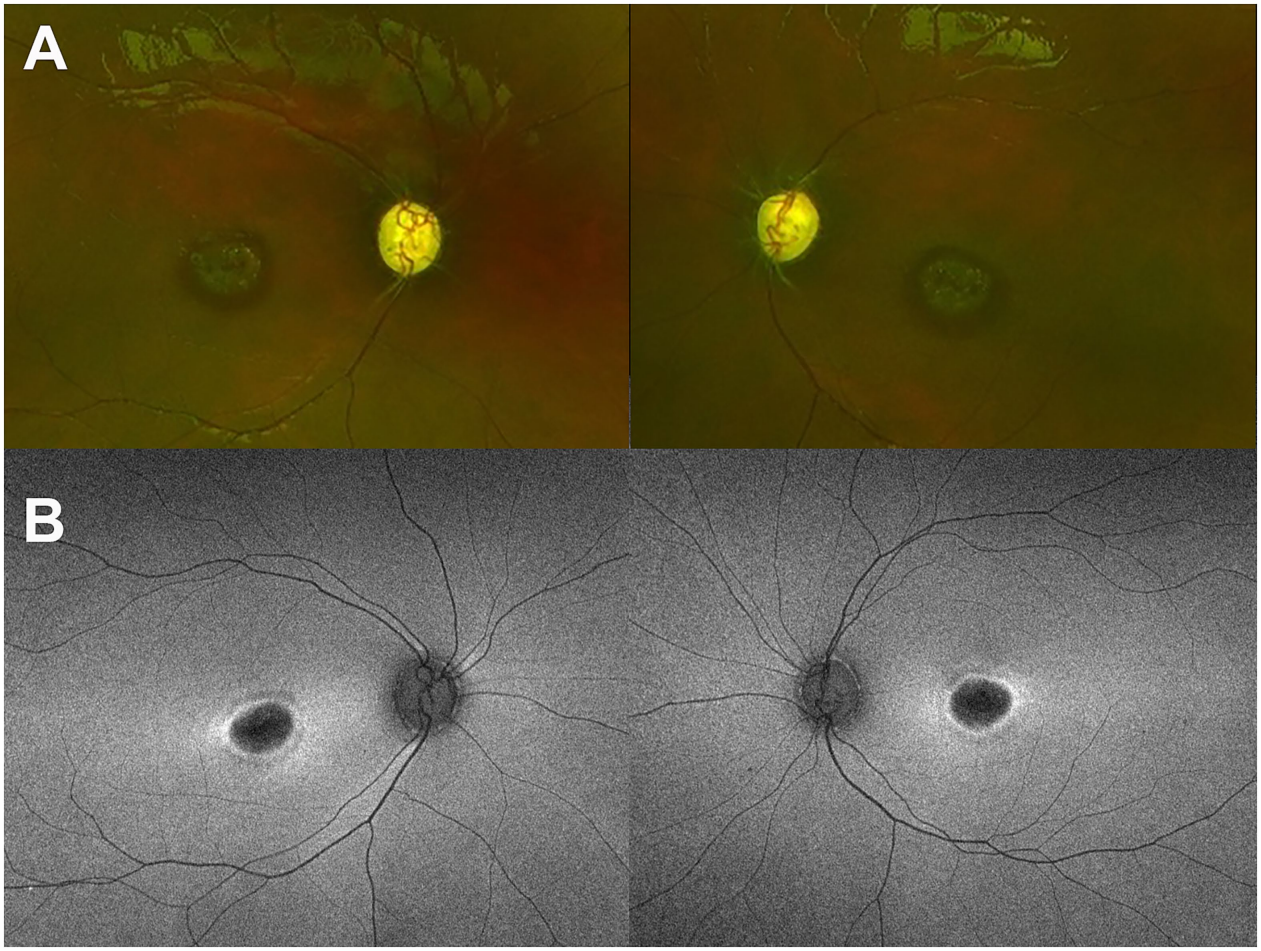
**(A)** Color fundus photographs and **(B)** autofluorescence images of the right and left eyes of Case 1 at the last follow-up visit.

**Figure 2:**
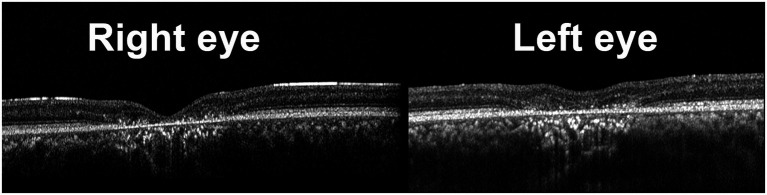
Optical coherence tomography (OCT) scans of the right and left macula of Case 1.

She continued to be followed with annual ophthalmologic evaluations while additional genetic testing was pursued. At her most recent ophthalmologic evaluation at 17 years of age, she demonstrated severe bilateral visual impairment; visual acuity was relatively stable at 1/80 in the right eye and 1/100 in the left eye. Nystagmus and strabismus were also stable, with an exotropia measuring 35 PD in primary gaze. Posterior segment examination demonstrated optic disc pallor bilaterally and central macular atrophy with pigmentary changes in both eyes (Figure 1A). Fundus autofluorescence imaging showed central macular hypo-autofluorescence with surrounding hyper-autofluorescence bilaterally (Figure 1B). Spectral-domain optical coherence tomography (OCT) demonstrated outer retinal atrophy with loss of the ellipsoid zone, outer nuclear layer, and outer plexiform layer (Figure 2).

#### Case 2

Case 2 is a 7-year-old male, the younger brother of Case 1, who was delivered at 38 weeks’ gestation via cesarean section with a birth weight of 6 lbs 11 oz. Microcephaly was first noted at approximately 6 months of age in the setting of delayed developmental milestones.

Neurologic evaluation and imaging were performed at outside institutions between 6 and 12 months of age. Skull radiographs and head CT were reported as normal aside from microcephaly. Electroencephalography was normal. Brain MRI with and without contrast did not demonstrate significant structural abnormalities.

He was first evaluated in our pediatric neuro-ophthalmology clinic at 2 years 8 months of age. Visual acuity was recorded as poor fixation in either eye monocularly, although he was able to fixate and follow a 3-inch toy at 1 foot binocularly. He had weak pupillary reactivity with no RAPD. He was unable to perform stereopsis testing. Ductions were full, and he had an esotropia measuring approximately 15 PD in primary gaze. He also had conjugate, pendular, horizontal nystagmus. Fundus examination revealed diffuse pallor and cupping of the optic nerves. The optic nerve was visualized via direct ophthalmoscopy through an undilated pupil and therefore detailed macular examination was not reported. He had no significant refractive error. Genetic testing was recommended.

At approximately 3 years of age, he was again evaluated by outside pediatric and neurology providers for developmental delay and was diagnosed with autism spectrum disorder. At that time, he demonstrated significant motor delay, was rolling or scooting, beginning to pull to stand, and spoke approximately four words. Additional diagnoses included hearing impairment and cryptorchidism.

At his most recent ophthalmologic evaluation at CHLA at 7 years of age, he could not perform quantitative visual acuity testing but was noted to have good fix and follow to a 2-inch toy at 2 feet. There was no RAPD, and sensory nystagmus and esotropia were stable or slightly improved. Posterior segment examination demonstrated diffuse optic disc pallor bilaterally and central macular atrophy in both eyes. He was unable to cooperate with ophthalmic imaging.

### Genetic testing

Both siblings underwent targeted genetic testing, including inherited retinal disease panels, without identification of a molecular diagnosis. The genes included in the ocular focused exome panel performed at CHLA are provided in Supplementary material. The siblings were evaluated by genetic counselors at CHLA at 16 and 6 years of age, respectively. Given the presence of early-onset optic atrophy, macular atrophy, and neurodevelopmental impairment similarly affecting the two siblings, clinical quad whole genome sequencing (WGS), including both parents, was ordered by the CHLA genetics team through Baylor Genetics. Our genetics team routinely performs WGS in patients with multi-systemic abnormalities and/or neurodevelopmental disorders because of the superior diagnostic yield and efficiency of WGS over exome sequencing (17, 18).

WGS identified compound heterozygous variants in the *COG5* gene in both siblings, with autosomal recessive inheritance. In both cases, a paternally inherited frameshift variant, c.1415dup (p.Gly474Trpfs*3), classified as pathogenic (ACMG/AMP Criteria (19): PVS1, PM2, PM3), and a maternally inherited deep intronic variant, c.417 + 4779A>G, classified as a variant of uncertain significance (ACMG/AMP Criteria (19): PM2, PM3, PP3), were detected *in trans*. Frameshift variants resulting in premature termination and predicted nonsense-mediated mRNA decay have been previously reported as a disease-causing mechanism in *COG5*-related CDG (7–9). The c.1415dup (p.Gly474Trpfs*3) variant is rare and was identified in 14/1,613,730 alleles in population studies without any homozygotes (gnomAD v4.1.0) (20). It was also reported in trans with another *COG5* pathogenic variant in an individual with congenital disorder of glycosylation, type IIi (13). The c.417 + 4779A>G variant occurs deep within an intron and is predicted to disrupt splicing by activating a cryptic splice acceptor site with a high SpliceAI score (0.98) (21, 22). This variant is rare and was only identified in 2/150,068 alleles in population studies without any homozygotes (gnomAD v4.1.0) (20). Given this evidence along with the knowledge that the c.417 + 4779A>G variant segregates with disease between the affected siblings and is present *in trans* with the pathogenic c.1415dup variant, a disease-causing role is strongly favored. Both variants were confirmed by Sanger sequencing.

## Discussion

This report describes two siblings with optic atrophy, central macular atrophy, sensory strabismus, and nystagmus, in the setting of neurodevelopmental delays who were diagnosed with COG5-related congenital disorder of glycosylation. There are few prior reports of optic nerve and retina involvement in individuals with this disorder. In Table 1, we have summarized the prior cases in the literature and compared them to the current reported cases with their genotypes and ophthalmologic and neurologic manifestations. One case series of three related individuals reported early-onset severe retinal degeneration with pigmentary retinopathy, retinal vessel attenuation, and optic atrophy (10). Electroretinogram was consistent with a cone-rod dystrophy. The authors performed a series of experiments demonstrating that *COG5* variants result in the aggregation of unfolded proteins, activating the unfolded protein response (UPR). This, in turn, leads to upregulation of PKR-like endoplasmic reticulum kinase (PERK), which is present on the inner segment of photoreceptors and induces DNA damage in the murine retina. This cascade of events is believed to result in the cone-rod dystrophy phenotype of the family reported and could also be contributory in our patients. However, in the aforementioned case series, the affected individuals had no neurocognitive impairment, unlike the two siblings reported herein.

**Table 1:** Cases of COG5-related congenital disorder of glycosylation reported in the literature, including the two cases in the current report, Genotypes and ophthalmologic and neurologic findings are provided.

First author and year of publication	Patient identity in manuscript	Genotype	Ophthalmologic findings	Neurologic findings
Wang X 2020 (9)	Only case	c.1290C>A (p.Y430X)/c.2077A>C (p.T693P)	Strabismus, macular atrophy, foveal hypoplasia	Developmental delay, intellectual disability
Tabbarah 2020 (10)	II-1	c.2327dupT (p.Ser777Glnfs*14)/c.95 T>G (p.Met32Arg)	Severe early onset cone-rod dystrophy with optic atrophy, nystagmus	Microcephaly; normal cognition and neuro-development
Tabbarah 2020 (10)	II-2	c.2327dupT (p.Ser777Glnfs*14)/c.95 T>G (p.Met32Arg)	Severe early onset cone-rod dystrophy with optic atrophy, nystagmus	Microcephaly; normal cognition and neuro-development
Tabbarah 2020 (10)	II-2	c.2327dupT (p.Ser777Glnfs*14)/c.95 T>G (p.Met32Arg)	Severe early onset cone-rod dystrophy with optic atrophy, nystagmus	Microcephaly; normal cognition and neuro-development
Wang YC 2024 (11)	Only case	c.2 T>G (start loss, paternal)/c.1826 T>C (uncertain significance, paternal)/c.463_467delAGTAAinsCT (frameshift, maternal)	Strabismus, optic atrophy	Hypotonia, global developmental delay
Granger 2026 (current report)	Case 1	c.1415dup (pathogenic frameshift)/c.417 + 4779A>G	Bilateral optic atrophy, central macular atrophy, nystagmus, strabismus	Global developmental delay, hypotonia, polymicrogyria
Granger 2026 (current report)	Case 2	c.1415dup (pathogenic frameshift)/c.417 + 4779A>G	Bilateral optic atrophy, central macular atrophy, nystagmus, strabismus	Microcephaly, global developmental delay, ASD

Genotypes and ophthalmologic and neurologic findings are provided.

ASD, autism spectrum disorder.

Foveal hypoplasia with fundus and OCT appearance similar to our cases were reported in a 4-year-old Chinese girl with COG5-congenital disorder of glycosylation who also demonstrated cognitive impairment (intelligence age 23.4 months), ataxia, and psychomotor delay, with normal brain MRI (9). Optic nerve appearance was not mentioned in this case. In contrast, a case report of an 11-year-old girl with COG5-congenital disorder of glycosylation described optic atrophy on examination and MRI without mention of macular pathology (11). Thus, our cases are unique in combining both optic nerve and macular atrophy with neurodevelopmental delays. It is unclear whether genotype differences may account for phenotypic variation. The variants in our siblings included one previously reported pathogenic frameshift variant; the affected individual in the literature was not noted to have any ophthalmic pathology, although detailed ophthalmologic examination results were not reported (13). The second variant, a deep intronic variant, has not been previously reported in the literature. In the previously reported cases of patients with COG-CDG who had optic nerve and/or macular pathology (Table 1), compound heterozygous loss of function variants were identified in one child with optic atrophy and global developmental delay, without reported macular abnormalities (11). One child with macular (but not optic nerve) atrophy and intellectual disability (9), and three siblings with pigmentary retinopathy and optic atrophy but normal neurodevelopment were compound heterozygotes for a loss of function and missense variant (10).

Phenotypic variability between siblings despite identical *COG5* variants was also observed. Case 1 demonstrated polymicrogyria on brain MRI, whereas Case 2 exhibited microcephaly without a structural cortical malformation. Autism spectrum disorder was diagnosed in Case 2 but not Case 1. Similar variability has been reported in other individuals with COG5-CDG and related CDG disorders and emphasizes the heterogeneous expressivity of this condition (8, 9, 23, 24).

The coexistence of optic atrophy and macular pathology presents a diagnostic challenge in pediatric neuro-ophthalmology and spans a wide differential diagnosis (12). In both siblings, the early onset and progressive severity of visual impairment, combined with developmental delay and additional neurologic features, prompted extensive evaluation across multiple institutions. Despite comprehensive panel-based genetic testing, a molecular diagnosis was not established until whole genome sequencing was pursued. Whole genome sequencing proved diagnostically informative in this family by identifying a deep intronic *COG5* variant predicted to alter splicing, which would not have been detected by targeted panel or standard exome sequencing (14–16, 21, 22).

Strengths of this report include clinical quad WGS with confirmed parental inheritance in both siblings and complete ophthalmic characterization with examinations by pediatric neuro-ophthalmology and retina specialists and ophthalmic imaging (where possible). This provided the opportunity to compare phenotypic variability within a single genotype. A limitation is the lack of imaging and quantitative visual acuity data from initial ophthalmologic examinations outside our institution, which limits determination of whether the optic atrophy and macular changes represent static or progressive findings.

Establishing a diagnosis of COG5-CDG has important clinical implications. Although disease-specific therapies are not currently available, molecular diagnosis allows for better educational guidance, coordination of multidisciplinary care, and accurate genetic counseling regarding autosomal recessive inheritance with a 25% recurrence risk (16). For neurologists and ophthalmologists, recognition of retinal and optic nerve involvement in association with developmental delays and structural brain abnormalities in COG5-CDG may prompt earlier consideration of this diagnosis in children with this combination of findings. Furthermore, *COG5* may be a candidate for inclusion on inherited retinal disorder panels. These cases expand the phenotype associated with COG5-related congenital disorder of glycosylation and support the role of WGS in understanding complex neuro-ophthalmologic presentations.

## Conclusion

This case report expands the known phenotypic spectrum of this rare condition by demonstrating optic atrophy and macular pathology alongside neurodevelopmental delay. The family’s diagnostic course, resolved only through whole genome sequencing, underscores the limitations of targeted panel-based testing. For clinicians, these cases highlight the importance of considering COG5-CDG in children presenting with unexplained optic atrophy and macular disease in the context of developmental delay and support early integration of genome-wide sequencing in such evaluations. Although no disease-modifying therapies currently exist, achieving a molecular diagnosis can enable accurate genetic counseling, coordinated multidisciplinary care, and a framework for future therapeutic consideration as treatments for CDG disorders continue to evolve.

## Data Availability

The original contributions presented in the study are included in the article/Supplementary material, further inquiries can be directed to the corresponding author.
