# Supplementary material for: COG5-congenital disorder of glycosylation diagnosed by whole genome sequencing in siblings with unexplained optic atrophy, macular atrophy, and developmental delay: case report
Source: Front Neurol. 2026 Jun 1;17:1840802. doi: 10.3389/fneur.2026.1840802 (PMC13265530; doi:10.3389/fneur.2026.1840802)
Supplement: Supplementary file 1 [file Data_Sheet_1.PDF]

CHILDREN'S HOSPITAL LOS ANGELES (CHLA) OCULAR DISEASE FOCUSED EXOME GENE LIST
The ocular disease focused exome test involves analysis of exome sequencing data for single nucleotide variants (SNVs) or small deletions and insertions (<10 bp) in a predefined set of 309 genes associated with ocular disease (see below). These include genes known to be associated with ocular disease as the only feature, as well as genes associated with certain syndromes for which ocular disease is a commonly observed feature in affected individuals.

The following nuclear genes were evaluated for substitutions and small indels (for each gene the percentage of the coding region covered at the minimum of 10X is provided in parentheses):

ABCA4(100%), ABCC6(97.37%), ABHD12(100%), ACBD5(99.71%), ACO2(95.8%), ADAM9(100%), ADAMTS18(100%), ADGRA3(97.92%), ADGRV1(100%), ADIPOR1(100%), AFG3L2(99.24%), AGBL5(100%), AHI1(100%), AHR(100%), AIPL1(100%), ALMS1(100%), ALPK1(100%), AP3B1(100%), AP3D1(98.84%),ARHGEF18(100%), ARL2BP(100%), ARL3(100%), ARL6(99.18%), ARSG(98.59%), ASRGL1(100%), ATF6(100%), ATXN7(100%), AUH(100%), BBIP1(100%), BBS1(100%), BBS10(100%), BBS12(100%), BBS2(100%), BBS4(100%), BBS5(100%), BBS7(100%), BBS9(99.47%), BEST1(100%), BLOC1S3(100%), BLOC1S6(100%), C1QTNF5(100%), CA4(100%), CABP4(100%), CACNA1F(100%), CACNA2D4(100%), CAPN5(100%), CC2D2A(99.9%), CCT2(100%), CDH23(100%), CDH3(100%), CDHR1(100%), CEP164(100%), CEP19(100%), CEP250(100%), CEP290(99.99%), CEP78(100%), CERKL(100%), CFH(100%), CHM(98.68%), CIB2(100%), CISD2(88%), CLCC1(99.47%), CLN3(100%), CLN5(87.5%), CLN6(100%), CLN8(100%), CLRN1(100%), CLUAP1(100%), CNGA1(100%), CNGA3(100%), CNGB1(100%), CNGB3(100%), CNNM4(100%), COL11A1(100%), COL11A2(100%), COL2A1(100%), COL9A1(99.63%), COL9A2(100%), COL9A3(100%), CRB1(100%), CRX(100%), CSPP1(99.13%),CTNNA1(99.83%), CTSD(100%), CYP1B1(100%), CYP4V2(100%), DHDDS(100%), DHX38(100%), DMD(100%), DNAJC30(100%), DRAM2(97.17%), DTHD1(100%), DTNBP1(100%), EFEMP1(100%), ELOVL1(100%), ELOVL4(100%), EMC1(98.56%), ESPN(97.7%), EXOSC2(100%), EYS(100%), FAM161A(100%), FLVCR1(100%), FOXC1(100%), FOXF2(100%), FSCN2(100%), FZD4(100%), GDF6(100%), GNAT1(100%), GNAT2(100%), GNB3(100%), GNPTG(100%), GPR143(97.65%), GPR179(100%), GRK1(100%), GRM6(100%), GUCA1A(100%), GUCA1B(100%), GUCY2D(100%), HGSNAT(100%), HK1(100%), HMCN1(100%), HMX1(100%), HPS1(100%), HPS3(100%), HPS4(99.06%), HPS5(100%), HPS6(100%), IDH3B(100%), IFT140(100%), IFT172(100%), IFT27(100%), IFT81(98.63%), IMPDH1(100%), IMPG1(100%), IMPG2(100%), INPP5E(100%), INVS(100%), IQCB1(98.81%), ITM2B(100%), JAG1(100%), KCNJ13(100%), KCNV2(100%), KIAA1549(98.15%), KIF11(100%), KIZ(100%), KLHL7(100%), LAMA1(100%), LCA5(100%), LOXL3(100%), LRAT(100%), LRIT3(100%), LRMDA(100%), LRP5(100%), LTBP2(100%), LYST(99.99%), LZTFL1(100%), MAK(100%), MAPKAPK3(100%), MERTK(100%), MFN2(100%), MFRP(100%), MFSD8(100%), MIR204(100%), MITF(99.15%), MKKS(81.82%), MKS1(100%), MLPH(100%), MTPAP(100%), MTTP(100%), MVK(100%), MYO5A(99.04%), MYO7A(100%), MYOC(100%), NBAS(100%), NDP(100%), NDUFS1(100%), NEK2(99.8%), NEUROD1(100%), NMNAT1(100%), NPHP1(100%), NPHP3(100%), NPHP4(100%), NR2E3(100%), NR2F1(100%), NRL(100%), NYX(100%), OAT(100%), OCA2(100%), OFD1(100%), OPA1(100%), OPA3(100%), OPN1LW(83.33%), OPN1MW(0%), OPN1SW(100%), OPTN(100%), OTX2(100%), P3H2(100%), PANK2(100%), PAX2(95.45%), PAX6(99.42%), PCDH15(99.15%), PCYT1A(100%), PDE6A(100%), PDE6B(100%), PDE6C(100%), PDE6G(100%), PDE6H(100%), PDZD7(99.42%), PEX1(100%), PEX2(100%), PEX7(100%), PGK1(100%), PHYH(100%), PITPNM3(100%), PITX2(100%), PLA2G5(100%), PLK4(100%), PNPLA6(100%), POC1B(100%), POC5(100%), POLG(100%), POMGNT1(100%), PPT1(100%), PRCD(100%), PRDM13(100%), PROM1(100%), PRPF3(100%), PRPF31(100%), PRPF4(100%), PRPF6(100%), PRPF8(100%), PRPH2(100%), PRPS1(100%), RAB27A(100%), RAB28(100%), RAX2(100%), RB1(96.21%), RBP3(100%), RBP4(100%), RCBTB1(100%), RD3(100%), RDH11(100%), RDH12(100%), RDH5(100%), REEP6(100%), RGR(100%), RGS9(100%), RGS9BP(100%), RHO(100%), RIMS1(99.33%), RLBP1(100%), ROM1(100%), RP1(78.12%), RP1L1(100%), RP2(100%), RP9(91.25%), RPE65(100%), RPGR(99.55%), RPGRIP1(100%), RPGRIP1L(98.51%), RS1(100%), RTN4IP1(100%), SAG(100%), SAMD11(96.03%), SDCCAG8(100%), SEMA4A(100%), SLC24A1(100%), SLC24A5(100%), SLC25A46(100%), SLC38A8(100%), SLC45A2(100%), SLC7A14(100%), SNRNP200(100%), SPATA7(100%), SPG7(97.78%), SPP2(100%), TEAD1(100%), TEK(100%), TIMM8A(100%),

TIMP3(100%), TMEM126A(100%), TMEM216(100%), TMEM237(100%), TOPORS(100%), TPP1(100%), TREX1(100%), TRIM32(100%), TRNT1(100%), TRPM1(100%), TSPAN12(100%), TTC8(100%), TTLL5(100%), TTPA(100%), TUB(100%), TUBB4B(100%), TUBGCP4(100%), TUBGCP6(100%), TULP1(100%), TYR(100%), TYRP1(100%), UNC119(100%), USH1C(100%), USH1G(100%), USH2A(100%), VCAN(100%), WDPCP(98.99%), WDR19(100%), WFS1(100%), WHRN(100%), ZNF408(100%), ZNF423(96.43%), ZNF513(100%)

The mitochondrial genome was also evaluated for substitutions and small indels. The percentage of bases with at least 100x coverage was 100%.
In total, 142877311 reads (100 bp) were generated and aligned to both the human reference genome and mitochondrial genome (GRCh38), generating a mean coverage of 152x per base within the RefSeq protein-coding exons and splice junctions for the nuclear genome and an average coverage of 9972x for mitochondrial genome.
